# Role of Gut Microbiota in the Aetiology of Obesity: Proposed Mechanisms and Review of the Literature

**DOI:** 10.1155/2016/7353642

**Published:** 2016-09-15

**Authors:** Muhammad Jaffar Khan, Konstantinos Gerasimidis, Christine Ann Edwards, M. Guftar Shaikh

**Affiliations:** ^1^Institute of Basic Medical Sciences, Khyber Medical University, Phase V Hayatabad, Peshawar, Khyber Pakhtunkhwa, Pakistan; ^2^Human Nutrition, School of Medicine, Dentistry and Nursing, College of Medical Veterinary and Life Sciences, University of Glasgow, Level 3, New Lister Building, Glasgow Royal Infirmary, 10-16 Alexandra Parade, Glasgow G31 2ER, UK; ^3^Department of Endocrinology, Royal Hospital for Children, 1345 Govan Rd, Govan, Glasgow G51 4TF, UK

## Abstract

The aetiology of obesity has been attributed to several factors (environmental, dietary, lifestyle, host, and genetic factors); however none of these fully explain the increase in the prevalence of obesity worldwide. Gut microbiota located at the interface of host and environment in the gut are a new area of research being explored to explain the excess accumulation of energy in obese individuals and may be a potential target for therapeutic manipulation to reduce host energy storage. Several mechanisms have been suggested to explain the role of gut microbiota in the aetiology of obesity such as short chain fatty acid production, stimulation of hormones, chronic low-grade inflammation, lipoprotein and bile acid metabolism, and increased endocannabinoid receptor system tone. However, evidence from animal and human studies clearly indicates controversies in determining the cause or effect relationship between the gut microbiota and obesity. Metagenomics based studies indicate that functionality rather than the composition of gut microbiota may be important. Further mechanistic studies controlling for environmental and epigenetic factors are therefore required to help unravel obesity pathogenesis.

## 1. Introduction

*Initial Evidence of the Role of Gut Microbiota in Obesity*. The worldwide increase in obesity has prompted researchers to investigate its aetiology which is multifactorial, involving environmental, dietary, lifestyle, genetic, and pathological factors. Although the gut microbiota were already established as a metabolic organ that could ferment nondigestible dietary components (particularly nondigested carbohydrates) to generate short chain fatty acids (SCFA), their role as a significant environmental factor affecting host adiposity through an integrated host signalling pathway was explored in 2004 by Bäckhed and colleagues [1]. This breakthrough evidence suggested that the gut microbiota induced adiposity by stimulating hepatic* de novo* lipogenesis and triglyceride storage through carbohydrate response element binding protein (ChREBP) and sterol response element binding protein 1 (SREBP1) and by suppressing fasting induced adipocyte factor (*fiaf*) which is an inhibitor of adipocyte lipoprotein lipase [1]. The same group proposed that this intestinal “high-efficiency bioreactor” in certain individuals might promote energy storage (obesity), whereas a low-efficiency reactor would promote leanness due to lesser energy harvest from carbohydrate fermentation [2]. Differences in the gut microbiota between obese and lean people were therefore worthy of further exploration.

Subsequent studies conducted by the same group suggested that although gut microbiota communities were shared between mothers and offspring regardless of* ob* genotype in genetically obese leptin deficient C57BL/6J* ob/ob* mice and lean mice (*ob/+ *and +/+ wild-type siblings) fed similar polysaccharide rich diets, the* ob/ob* mice had reduced relative abundance of Bacteroidetes (by 50%) and a proportional increase in Firmicutes regardless of kinship [3]. A higher Firmicutes to Bacteroidetes ratio was therefore suggested to be associated with increased energy harvest from food facilitated by the gut microbiota. However, no evidence was presented to show increased expression of genes related to bacterial metabolic activity and how this could be affected by diet and lifestyle nor whether these changes could also be seen in humans.

Turnbaugh et al. (2006) used whole genome shotgun metagenomic and microbiota transplantation studies to investigate the mechanisms [4]. They observed a high Firmicutes rich microbiome in* ob/ob* mice clustered together (in nonmetric multidimensional scale plot), richer in enzymes for degradation of polysaccharides, higher faecal acetate and butyrate, and less stool energy loss than in lean mice. Transplantation of gut microbiota from* ob/ob* mice or lean mice to germ-free mice resulted in obese (high Firmicutes) or lean (high Bacteroidetes) gut microbiome in the recipients. Obese microbiome recipients had higher percentage body fat despite similar food intake.

In a human study [5], obese adults were randomised onto fat or carbohydrate restricted diets and followed up for one year. Despite marked interpersonal variations in gut microbiota diversity, obese people had a lower relative abundance of Bacteroidetes and a higher relative abundance of Firmicutes before the restricted calorie intake. However, over the period of follow-up, the relative abundance of Bacteroidetes significantly increased while that of Firmicutes significantly reduced. Increased Bacteroidetes was significantly positively correlated with percentage weight loss and not the caloric content of diet [5]. This suggested that the gut microbiota restructured, changing their metabolic priorities to support coexistence in a changed environment. However, this study did not explore the same relationship in a parallel lean group to see whether the lean phenotype had the same response to dietary intervention.

Further evidence suggested the presence of the gut microbiota was necessary for development of obesity as germ-free mice were resistant to obesity even when they consumed more calories from normal chow or a high fat Western-type diet compared with CONV mice [13]. However, this idea was challenged in a later study by Fleissner et al. (2010) [14] who found that germ-free mice on a high fat diet gained significantly more weight and body fat and had less energy expenditure than lean CONV mice. Additionally, intestinal* fiaf* increased in HF and WD fed GF mice compared to CONV mice but not in the systemic circulation [14].

Several possible mechanisms were proposed to explain the impact of structural and functional differences in gut microbiota in lean and obese individuals that may contribute to host adiposity and whether an obese phenotype is transmissible by transplantation of gut microbiota. However, most of these studies were conducted in experimental animals which exhibited different anatomical, physiological, and bacterial colonisation patterns from humans. Several human and animal based studies have now revealed controversial evidence attributing differences in gut microbiota to the differences in diet [15–17] while others suggested no such association [18].

## 2. Proposed Mechanisms for the Role of Gut Microbiota in Obesity

The gut microbiota can be regarded as a “microbial organ” contributing to a variety of host metabolic processes from digestion to modulation of gene expression. The differences in gut microbiota between lean and obese animals or human subjects suggest a link between gut microbiota and energy homeostasis although there is still some debate as to whether these differences are causally related to an obese or lean phenotype. Various mechanisms have been suggested to link gut microbiota with obesity-genesis and other metabolic disorders (Table 1). However, it is still unclear how these mechanisms interact to influence the overall metabolic status of an individual.

### 2.1. Energy Harvest from Diet (Short Chain Fatty Acids)

Dietary polysaccharides and proteins that escape digestion in the small intestine are fermented in the colon by the gut microbiota into SCFA mainly acetate propionate and butyrate. The amount of energy harvested is hypothesised to be influenced by the composition of the gut microbiota [2]. It has been estimated that up to 10% of daily energy requirement and up to 70% of energy for cellular respiration for the colonic epithelium may be derived from SCFA. Chronic excess energy harvest may cause long term increased fat accumulation in the body [72].

To a greater extent, there is a general agreement from many studies that the obese phenotype is associated with excess SCFA in caecal and faecal samples in animal and human studies compared with the nonobese (Table 2). However, there is considerable disagreement and controversy over the population of the gut microbiota that may be associated with increased caecal or faecal SCFA measured (Table 3). Whether increased SCFA production results in increased energy harvest from the diet in obese phenotypes depends on several factors such as substrate availability, gut transit, mucosal absorption, gut health, production by the gut microbiota, and symbiotic relationships between different groups of gut microbiota [66]. Based on the equation derived by Livesey (1990), approximately 50% (2 kcal/g) of the energy derived from glucose is available after fermentation. The net amount of energy derived will therefore vary depending upon the amount of indigestible carbohydrate available for fermentation [73].

The obese phenotype in animals is associated with higher total caecal SCFA, acetate, and butyrate and higher expression of bacterial genes responsible for polysaccharide metabolism [4]. Increased efficiency in production of SCFA in obesity might also result from crosstalk between different species and genera to maintain their growth and population. Absorption of these SCFA, coupled with other lifestyle and environmental factors may result in excess energy storage and obesity. It is not clear whether this is an effect of substrate (i.e., carbohydrates) or the population of specific gut microbiota associated with increased SCFA production, absorption, and storage in adipose tissues and liver. The results are largely confounded by the study settings, lifestyle, and environmental factors of the study subjects.

### 2.2. Gut Microbiota and Fasting Induced Adipocyte Factor

Fasting induced adipocyte factor or angiopoitein-like protein 4 (*Fiaf*/ANGPTL4) is a target gene for peroxisome receptor activated proteins (PPARs) and is produced by large intestinal epithelial cells and the liver.* Fiaf*/ANGPTL 4 inhibits lipoprotein lipase (LPL) which causes accumulation of fat in peripheral tissues. Inhibition of* fiaf* by the gut microbiota with a resultant increase in LPL may be one mechanism for gut bacterial induced host adiposity [1]. This is further supported by studies on GF mice, genetically deficient in* fiaf *genes (*fiaf* −/−). Lack of the* fiaf* gene causes disinhibition of LPL which leads to deposition of up to 60% higher epididymal fat compared to germ-free wild-type littermates expressing fiaf genes (*fiaf *+/+).* fiaf*/ANGPTL4 is therefore involved in the regulation of fat storage mediated by the gut microbiota. Controlled manipulation of the gut microbiota may alter the expression of this hormone [74]. Normal weight SPF C57B/6J mice were fed either with high fat (20%) diet or high fat diet supplemented with probiotic* Lactobacillus paracasei* F19 for 10 weeks. Compared to the nonsupplemented group, plasma* fiaf*/ANGPTL4 was upregulated in the* Lactobacillus paracasei* F19 supplemented group with significantly elevated plasma VLDL but no change in other lipoproteins. In another study,* Lactobacillus paracasei* F19 and* Bifidobacterium lactis* BB12 were found to upregulate ANGPTL4 in the colon carcinoma HCT116 cell line in a dose and time dependent manner while* Bacteroides thetaiotaomicron* had no effect [74]. In the same study, the authors fed germ-free NMRI mice with normal chow and exposed them to F19. They found an increasing trend of ANGPTL4 in the serum after 2 weeks of colonisation, while the effect was not observed with heat killed F19 [74]. This study suggested that manipulation of expression of* fiaf*/ANGPTL4 is dependent on the gut microbiota and future interventional studies on weight management can be based on modification of ANGPTL4 by manipulating the gut microbiota.

Whether the increase in levels of* fiaf* in systemic circulation and the subsequent suppression of LPL and fat storage is associated with a change in gut microbiota has been questioned in some studies as there was no difference in* fiaf* in serum of GF and conventionally raised mice [14]. GF and CV mice were fed a low fat diet (LF), high fat diet (HF), and commercial high fat Western diet (WD). GF mice gained more weight and body fat than CV mice on HF and vice versa on WD. Although intestinal* fiaf*/ANGPTL4 was high in GF mice on HF and WD, circulating levels of fiaf did not change significantly compared to CV mice. The gut microbiota changed differently with HF and WD in CV mice. These observations suggested that diet affects the type of gut microbiota in the gut and that fiaf does not play a major role in peripheral fat storage as mentioned by other studies.

### 2.3. Gut Microbiota and Fatty Acid Oxidation

The gut microbiota are thought to reduce muscle and liver fatty acid oxidation by suppressing adenosine monophosphate kinase (AMPk), an enzyme in liver and muscle cells that acts as a fuel gauge monitoring cellular energy status. Inhibition of AMPk results in reduced muscle and liver fatty acid oxidation ultimately leading to excess fatty acids storage in these tissues [1].

Phosphorylated AMPk inhibits the formation of malonyl CoA via acetyl CoA carboxylase. Inhibition of malonyl CoA causes disinhibition of carnitine palmitoyltransferase-1 (Cpt-1) which in turn catalyses the rate limiting step in the entry of long chain fatty acyl-CoA into mitochondria for fatty acid oxidation [75]. Increased fatty acid oxidation is associated with enhanced cellular energy status coupled with glycogen level reduction and increased insulin sensitivity [75].

Germ-free mice have a consistently raised level of phosphorylated acetyl CoA carboxylase (Acc) and carnitine palmitoyltransferase-1 (Cpt-1) activity in gastrocnemius muscles and raised AMPk in liver and skeletal tissue compared to CONV mice [13, 76]. This effect has also been observed with high calorie diet suggesting that enhanced or suppressed muscle fatty acid oxidation is dependent on the presence or absence of gut microbiota. The gut microbiota may therefore influence storage of peripheral adipose tissue and hence host adiposity by inhibiting fatty acid oxidation.

### 2.4. Gut Microbiota and Bile Acids Circulation

**Figure 1:**
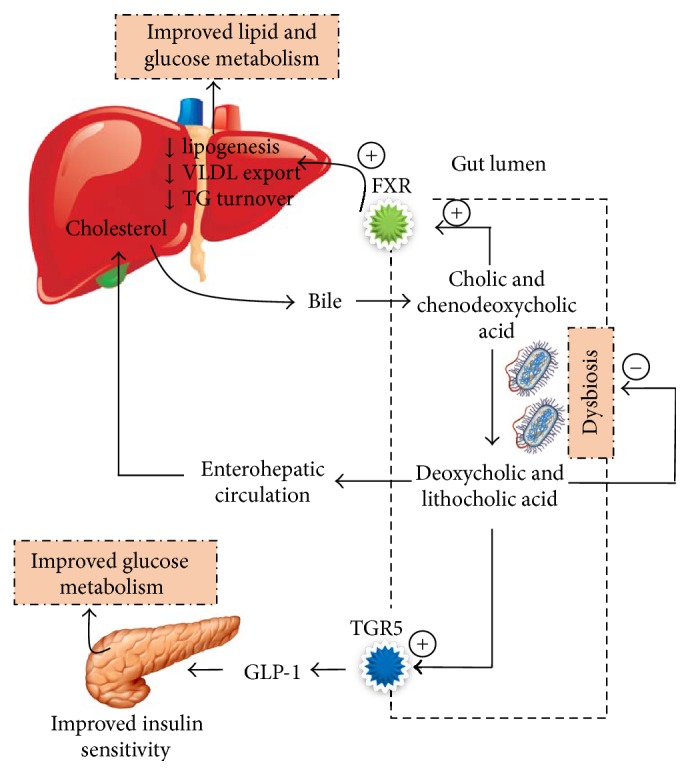
Modulation of bile acid circulation by gut microbiota and its effect on glucose metabolism. Concept adapted from [6–8]. TGR5: G protein coupled receptor 5, VLDL: very low density lipoprotein, TG: triglycerides, GLP-1: glucagon like peptide-1, and FXR: farnesoid x receptor.

Primary bile acids (cholic and chenodeoxycholic acids) are ligands for the farnesoid x receptor (FXR) which plays a key role in the control of hepatic* de novo* lipogenesis, very low density lipoprotein (VLDL) triglyceride export, and plasma triglyceride turnover leading to improved lipid and glucose metabolism [6]. By binding to FXR in ileal cells, bile acids are able to stimulate the expression of genes (Asbt, IBABP, and Ost *α*/*β*) which help in absorption, intracellular transport, and systemic transport of bile acids into the liver by enterohepatic circulation (Figure 1). Study on germ-free and FXR deficient mice suggests that the expression of genes responsible for the uptake, transport, and export of bile acids into circulation after ileocaecal resection is dependent on gut microbiota [19]. Primary bile acids entering the large intestine are converted to secondary bile acids (deoxycholic and lithocholic acids) by gut microbiota. Secondary bile acids are ligands for G protein coupled receptor 5 (TGR5) which helps in glucose homeostasis by stimulating the expression of glucagon like peptide-1 (GLP-1) and reduces serum and hepatic triglyceride levels [7, 8]. Gut microbiota may therefore affect host hepatic adiposity by altering bile acid circulation via FXR and TGR5 mechanisms. However, it is also suggested that bile acids may reciprocally cause dysbiosis through their bactericidal activity by damaging the microbial cell membrane phospholipid [77]. Furthermore, high saturated fat but not polyunsaturated fat promotes the expansion of pathobionts such as* Bilophila wadsworthia* and activates proinflammatory markers such as IL-10 causing experimental colitis [78].

### 2.5. Gut Microbiota and Changes in Satiety (Gut-Neural Axis)

**Figure 2:**
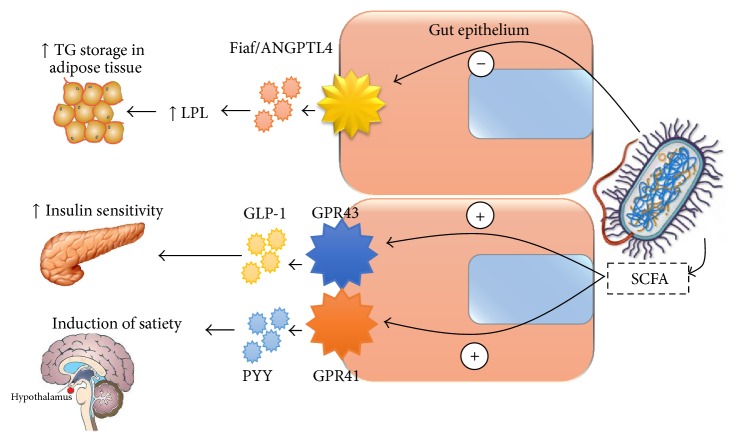
Proposed mechanism of the changes in gut hormonal axis by gut microbiota. TG: triglycerides, LPL: lipoprotein lipase, Fiaf: fasting induced adipocyte factor, ANGPTL-4: angiopoitein-like protein-4, GLP-1: glucagon like peptide-1, GPR43 and GPR41: G protein coupled receptors 43 and 41, PYY: peptide YY, and SCFA: short chain fatty acids. Minus sign indicates inhibitory effect; plus sign indicates stimulatory effect.

The gut microbiota, through production of SCFA, may affect host energy metabolism and development of obesity by changing the hormonal milieu in the intestine and other visceral organs (Figure 2). Glucagon like peptide-1 (GLP-1) plays a key role in regulating communication between the nutritional load in the gut lumen and peripheral organs such as brain, liver, muscle, and adipose tissue by postprandial increases in satiety, gut transit time, and incretin induced insulin secretion [79]. Secretion of GLP-1 is decreased in obesity secondary to weight gain which causes insulin resistance independent of circulating level of fatty acids [79]. The gut microbiota regulate GLP-1 by influencing the expression of its precursor, proglucagon, and increasing GLP-1 positive enteroendocrine L-cell in the gut [80]. Dietary fibres (nondigestible and fermentable fibres), as well as SCFA, have been shown to increase GLP-1 secretion in both human [81] and animal studies [82]. Mice lacking receptors for the attachment of SCFA (GPR43 and GPR41 deficient mice) showed* in vitro* and* in vivo* reduced GLP-1 secretion and impaired glucose tolerance [83].

SCFA including acetate, propionate, and butyrate act as ligands for the activation of G protein coupled receptors 43 and 41 (GPR41 and GPR43) which are expressed by gut epithelial cells, endocrine cells, and adipocytes. GPR43 in white adipose tissue act as sensors of postprandial energy excess and regulate energy expenditure and hence body energy homeostasis. GPR43 and GPR41 enhance insulin sensitivity and activate the sympathetic nervous system at the level of the ganglion to prevent excess energy deposition in adipose tissue and enhance energy expenditure in other tissues such as liver and muscles [22]. GPR43 deficient mice have metabolic abnormalities including excess fat accumulation. When treated with antibiotics or under germ-free conditions, these metabolic abnormalities reverse suggesting that the gut microbiota are key players in expression of these receptors [22]. Samuel et al. (2008) demonstrated that GF mice deficient in GPR41 genes remain lean compared with their wild type counterparts, although their body composition was not different [84]. They also showed that GPR41 stimulates the expression of the gut anorexigenic hormone, peptide YY (PYY), which in turn causes inhibition of gastric emptying, reduced intestinal transit time, increased energy harvest (in the form of caecal acetate and propionate), and increased hepatic lipogenesis [84].

Bifidobacteria are inversely associated with the development of fat mass, glucose intolerance, and bacterial lipopolysaccharide (LPS) in the blood via SCFA-induced stimulation of PYY and ghrelin. Intervention with prebiotics such as dietary fructans or oligofructose stimulates bifidobacterial growth [76] and reduces weight accompanied by increased PYY and reduced ghrelin consistent with a lower food intake in the prebiotics group [85]. Intervention with 16 g fructose/day or 16 g dextrin maltose/day for 2 weeks in a randomised control trial was associated with an increase in breath hydrogen (a marker of colonic fermentation) and increased production of PYY and GLP-1 [86].

Overall, this evidence suggests that alteration in the gut microbiota may affect hormonal status via GLP-1 and G protein coupled receptors. These hormonal changes bring a change in satiety, food intake, and overall metabolic status of an individual that could affect host adiposity. Whether this relationship is causal needs further investigation.

### 2.6. Gut Microbiota and Intestinal Permeability: Chronic Low-Grade Inflammation

**Figure 3:**
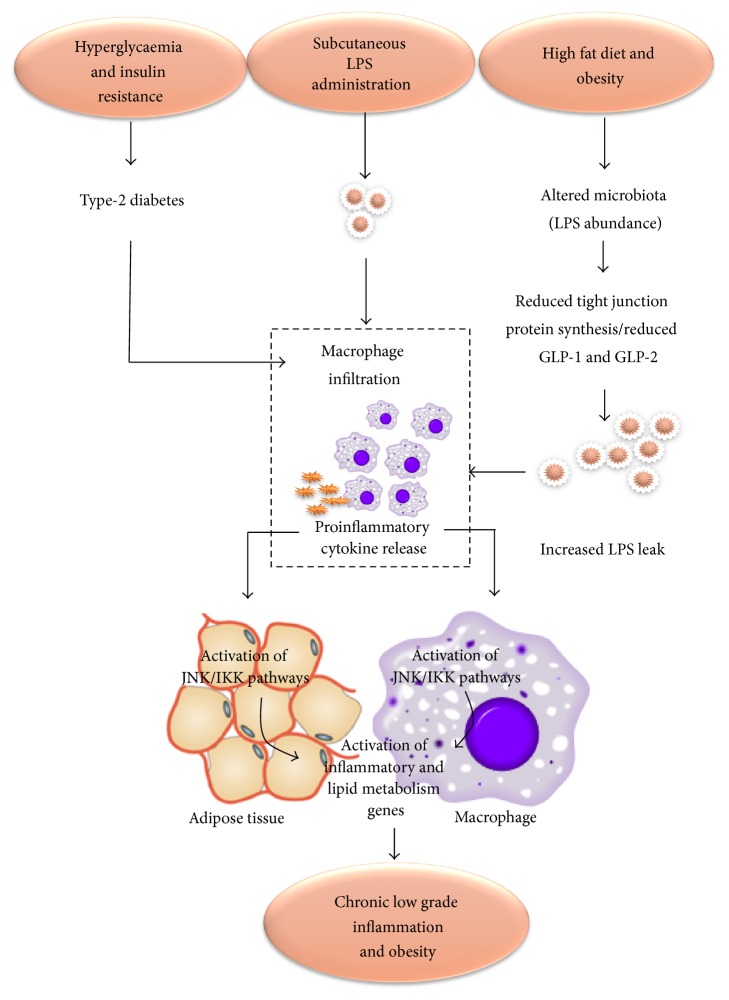
Proposed model for the role of LPS in generating inflammation and its relationship with obesity. Concept adapted from [9–12]. Altered mucosal barrier function due to reduced expression of glucagon like peptides 1 and 2 (GLP-1 and GLP-2) leads to altered mucosal function and reduced synthesis of tight junction proteins, Zonula Occludin-1 and Zonula Occludin-2 (ZO-1, ZO-2), increasing gut permeability. This allows LPS to enter the systemic circulation inducing the release of proinflammatory cytokines. Proinflammatory cytokines result in activation of a family of kinases JNK and IKK (inhibitor of NFkB kinase) that increase the expression of inflammatory and lipid metabolism genes. Subcutaneous administration of LPS, hyperglycaemia, and insulin resistance induces the same pathway by increasing the endoplasmic reticulum and mitochondrial stress. Type-2 diabetes, hyperglycaemia, and insulin resistance also cause macrophage infiltration and inflammatory cytokine release leading to the same process. HF: high fat diet [9–12].

Emerging evidence suggests close ties between metabolic and immune systems [11]. Obesity contributes to immune dysfunction by secretion of inflammatory adipokines from adipose tissues such as TNF-*α*, IL-6, and leptin [87]. Inflammatory adipokines induce carcinogenic mechanisms such as increased cellular proliferation and/or dedifferentiation that are potential risk factors for cancers such as colonic, oesophageal, and hepatocellular cancers. An example of this is the association of high levels of leptin with hepatocellular carcinoma [87]. Intra-abdominal adipose tissue secretes adipokines with atherogenic properties (IL-1, IL-6, TNF-*α*, and IFN-*α*) which increase the risk of cardiovascular diseases [88]. These proinflammatory cytokines also activate certain kinases, which in turn initiate the expression of inflammatory and lipogenic genes, ultimately increasing inflammation and adipogenesis in a loop fashion (Figure 3).

#### 2.6.1. Bacterial Lipopolysaccharide (LPS) and Inflammation

The gut microbiota may contribute to chronic low-grade inflammation and obesity via the absorption of bacterial LPS, an outer membrane component of Gram negative bacteria, which is increasingly recognized as a player in chronic low-grade inflammation, a hallmark of obesity.

Cani et al. (2007) demonstrated the link between LPS and metabolic disease by infusing bacterial LPS subcutaneously into germ-free mice for 4 weeks which produced the same level of metabolic endotoxemia as by high fat diet [9]. Furthermore, mice lacking functional LPS receptors were resistant to these changes. Feeding high fat diet to mice with mucosal immune dysfunction (Toll-Like Receptor-4 knockout mice) for 4 weeks resulted in two to three times increased systemic LPS levels in liver, adipose tissue and muscles, and higher body fat mass, termed as “metabolic endotoxemia” [9]. This inflammatory status was associated with lower* Bacteroides, Bifidobacterium* species, and* Eubacterium rectale-C coccoides* group [9]. Additionally, LPS stimulated markers of inflammation (e.g., plasminogen activator inhibitor 1 and tumour necrosis factor alpha) and oxidative stress (e.g., lipid peroxidation) in visceral adipose tissue via the CD14 receptor. Absence of CD14 in CD14 deficient* ob/ob* (CD14 −/−) mice has been shown to protect against diet induced obesity and inflammation in mouse models [10].

#### 2.6.2. Gut Barrier Integrity and Inflammation

Alteration in the gut microbiota is linked to changed gut barrier function [10] and may promote the release of bacterial endotoxins through damaged and leaky gut. Cani et al. (2007) showed a significant reduction in Bifidobacteria with high fat diet in male C57BL/6J mice [76]. Supplementation with oligofructose was shown to restore the Bifidobacteria population with improvement in gut barrier function evidenced by the expression of precursors of GLP-1, proglucagon mRNA, and decrease in endotoxemia [76]. No correlation was found between endotoxemia and other bacteria (*Lactobacilli/Enterococci*,* E. rectale/C. coccoides*,* Bacteroides*, and* sulphate reducing bacteria*) [76]. GLP-1 helps in the differentiation of mucosal cells into enteroendocrine L-cells, while GLP-2 helps in increased expression of mRNA for synthesis of tight junction proteins. These changes are associated with lower LPS in the blood suggesting increased integrity of the gut barrier function. In contrast treatment with antibiotics reduced inflammation by reducing the LPS-producing gut microbiota population, further elucidating the relationship between gut microbiota, LPS levels, and inflammation [10].

#### 2.6.3. High Fat Diet and Inflammation

The association of high fat diet with subclinical or clinical inflammation in obesity has been investigated in several studies and there is a clear evidence to suggest that consumption of high fat diet is associated with metabolic endotoxemia and 2-3-fold increase in bacterial LPS levels in the blood [9]. However, it is controversial whether this chronic low-grade inflammation is dependent on the gut microbiota. Cani et al. (2007) found a dramatic change in gut microbiota (reduced* Lactobacillus*,* Bacteroides/Prevotella,* and Bifidobacteria) of obese* ob/ob* mice fed high fat diet [76]. This was associated with an increase in gut permeability indicated by a reduced expression of Occludin and ZO-1 tight junction proteins.

In contrast, de la Serre et al. (2010) suggested that high fat diet induced intestinal inflammation in obese Sprague-Dawley rats may cause hyperphagia and obesity by impairing the regulation of food intake. However, changes observed in the gut microbiota were independent of lean and obese phenotype [15]. High fat diet for 8 or 12 weeks in Sprague-Dawley rats revealed two genetically distinct groups, diet induced obesity resistant (DIO-R) rats which were resistant to diet induced obesity and diet induced obesity prone (DIO-P) rats, which were prone to diet induced obesity on feeding high fat diet. DIO-P rats had significantly increased gut permeability, increased LPS levels, lower intestinal alkaline phosphatase (iAP) levels (which detoxifies LPS), and systemic inflammation (high Toll-Like Receptor-4/Mitogen Detector-2 protein immunoreactivity) compared to DIO-R [15]. Activation of TLR4 by LPS via MD-2 results in the production of an inflammatory cascade (IL-6 and TNF alpha) [89] ensuing metabolic endotoxemia. Mice with genetic deficiency of TLR4 do not develop diet induced obesity [34]. This series of changes associated with high fat diet inducing inflammation may alter food intake regulation and trigger hyperphagia, the mechanism of which is yet to be fully understood.

### 2.7. Gut Microbiota and Endocannabinoid Receptor System

Cannabinoid receptors 1 and 2 (CB1 and CB2) are G proteins activated by the endocannabinoid (eCB) system. The eCB system is composed of endogenous lipids and plays an important role in adipogenesis, as studied in genetically obese mice models. Two of the most widely studied lipids in the eCB system are N-arachidonoylethanolamine and 2-arachidonoylglycerol. The level of eCB components is inversely related to obesity and type-2 diabetes as both the conditions are associated with a higher tone of eCB system. Furthermore, the expression of CB1 and CB2 degrading enzymes (fatty acid amide hydrolase) is increased in adipose tissue of obese* ob/ob* mice compared with lean mice [10].

Bacterial LPS regulates the expression of cannabinoid receptors via the LPS receptor signalling system shown in both* in vitro *and* in vivo *studies [90]. This increased tone is represented by higher levels of the precursor enzymes N-acylphosphatidylethanolamine-selective phospholipase-D, CB1 mRNA, and increased eCB components in plasma or adipose tissue [90]. Using CB1 receptor antagonists in* ob/ob* obese mice with disrupted gut barrier and metabolic endotoxemia improves gut permeability and reduces body weight, compared with lean littermates [90]. The gut microbiota therefore regulate the activity of the eCB system and play an important role in host energy regulation.

A study by Geurts et al. (2011) in obese leptin resistant* db/db *mice suggested that the abundance of Gram negative bacteria, higher Firmicutes and Proteobacteria, and lower Bacteroidetes were correlated with upregulation of apelin and APJ expression. This was shown to be the result of direct action of bacterial LPS on the expression of apelin and APJ mRNA in obese diabetic mice through chronic low-grade inflammation [20]. These newly discovered adipokines are widely expressed in mammalian tissues. Apelin is a ligand for APJ, a G protein coupled receptor. Apelin/APJ system plays a key role in the cardiovascular system by acting on heart contractility, blood pressure, fluid homeostasis, vessel formation, and cell proliferation. Apelin also affects glucose homeostasis by acting through AMP kinase and nitric oxide (NO) dependent mechanisms [91]. Endocannabinoid system downregulates the expression of apelin and APJ mRNA in physiological conditions. In contrast, higher levels of apelin and APJ mRNA have been found in pathological conditions such as obesity and diabetes [20].

In summary, bacterial LPS increase the tone of eCB system and increase the expression of apelin/RPJ system in adipose tissue. However, how far gut microbiota population contribute to the actions of eCB and apelin/APJ and eCB in obesity is unknown. This has opened yet another area of interest in the role of gut microbiota in obesity.

## 3. Review of Animal Studies Relating Gut Microbiota with Obesity

The evidence from animal studies has thus far concentrated on studies which looked at the interplay of diet, gut microbiota, and metabolic changes (in energy balance, lipoproteins, cholesterol, etc.) in animal models such as wild-type mice, leptin deficient* ob/ob* mice, and Sprague-Dawley rats. Initial evidence suggesting a strong association of the gut microbiota with obesity was explored in a series of studies using germ-free and CONV mice. Components of gut microbiota acting as triggers in the development of obesity [40] and the emergence of diet induced obesity prone (DIO-P) mice and diet induced obesity resistant (DIO-R) mice fed on the same high fat diet [92] suggested that the peculiar compositional differences alter the host response to prioritise its metabolism towards increased energy harvest. Phylum level compositional differences in the relative proportions of the gut microbiota were therefore seen (Table 3) [1, 3, 4] and despite differences at species and genera level between studies, there is a general agreement on reduced diversity and richness of the gut microbiome in obese versus lean animals.

However the gut microbiota are located at the interface of environment and host. The effect of environmental factors particularly diet may therefore be highly significant and contribute to changes in the gut microbiota composition and function and ultimately their phenotype (obese or lean microbiome) [36]. Ingestion of high fat Western diets may play an important role in modifying the gut bacterial population which in turn alters the energy harvesting capability. This has been studied in various animal models such as GF/CONV mice and Sprague-Dawley rats [15, 17], leptin deficient* ob/ob* mice models [31], and immune deficient mice models (Toll-Like Receptor proteins deficient mice) [40] showing a tendency towards an increase in populations of Firmicutes and reduction in Bacteroidetes after feeding with high fat Western diet.

Furthermore, observations from studies on GF/CONV mice and Sprague-Dawley rats suggest that a high fat diet, especially HF Western diet, is associated with increased adiposity, reduced bacterial diversity [17], reduced number of* Bacteroides*, a relative increase in favour of Firmicutes [17], and higher jejunal alkaline phosphatase activity [31]. Moreover, high fat diet correlates with changes in inflammatory markers and oxidative stress [10] such as tumour necrosis factor alpha (TNF-*α*) and nuclear factor-kappaB (NF-kappaB), which play a major role in promoting inflammation [93], immune response, cellular proliferation, and apoptosis. In CONV mice, but not in germ-free mice, changes in the expression of these inflammatory markers in the intestine preceded weight changes and carried a strong positive correlation with high fat diet induced adiposity and markers of insulin resistance [32]. This suggests an interaction of high fat diet and enteric bacteria-promoting intestinal inflammation and insulin resistance prior to weight gain which is driven by the high fat diet.

Studies in leptin deficient* ob/ob* mice, genetically prone to obesity, indicated that although the obese phenotype is characterised by a particular set of gut microbiota, change in caloric load and diet redistributes the equilibrium that may be independent of the genotype or phenotype (obese or lean) [16]. Changes in gut microbiota composition may be attributed to the high fat diet rather than genetic propensity to obesity. Furthermore, shift towards higher Firmicutes to Bacteroidetes or the absence of gut microbiota may not be associated with the development of obesity [14]. The assertion that germ-free mice are protected from obesity was contradicted by Fleissner et al. (2010) where GF had a significantly higher body weight gain than CONV mice on high fat diet despite increased Firmicutes (specifically, Erysipelotrichaceae) at the expense of Bacteroidetes in CONV on a high fat diet and Western diet [14].

Faecal transplantation studies support the causal role of the gut microbiota in the aetiology of obesity. Transplantation of gut bacteria from obese human twins to lean mice caused not only obesity but also a higher number of genes involved in detoxification and stress response, biosynthesis of cobalamin, essential and nonessential amino acids, and gluconeogenic pathways. In contrast, animals with lean-transplanted microbiota exhibited genes capable of fermenting plant polysaccharides and producing butyrate and propionate [94]. Additionally, the mere presence of the gut microbiota in conventionally raised mice has been shown to result in higher levels of energy metabolites such as pyruvic, citric, fumaric, and malic acid and higher rate of clearance of cholesterol and triglycerides than in germ-free mice [95]. This suggests that the gut microbiota are essential for the characteristic pattern of metabolites in the gut of a species [96]. In postgastric bypass animals, gut microbiota transplanted from a postgastric bypass animals who lost weight after surgery were associated with weight loss and other metabolic changes in recipient obese mice with no surgery [97].

It is however interesting to observe that lean animals cohoused with obese cage mates are reported to develop obesity and obesity related microbiota and metabolism in some studies [17] but not others [94] although the microbiota and metatranscriptome of obese animals became similar to the lean phenotype suggesting a “functional transformation” [94]. As discussed above, the functional association of metabolic endotoxemia with gut microbiota was dependent on a high fat diet in the obese* ob/ob* animal model [10, 76]. However, these effects were later shown to be independent of obesity phenotype, as a high energy intake in lean C57BL/6J mice fed a high fat diet produced a 2-3-fold increase in plasma LPS compared to normal chow diet. Furthermore, the increase was blunted when the percentage intake of energy contributed by fat was reduced [98]. de Wit et al. (2012) showed that a high fat diet composed of palm oil (with more saturated fat) distinctly increased the Firmicutes to Bacteroidetes ratio in the gut compared to a diet high in fat-olive oil, high fat-safflower oil, and low fat-palm oil [35]. High fat-palm oil also stimulated expression of 69 genes related to lipid metabolism in the distal intestine suggesting an overflow of lipids to the distal small intestine resulted in enhanced lipid metabolism and changes in gut microbiota.

Several other studies suggested similar changes in gut microbiota and the presence of genes for lipid metabolism in animal models using different dietary regimens [37, 38, 99] (Table 2). Daniel et al. (2014) investigated composition and function of gut microbial ecology after 12 weeks of high fat diet (HF) or high carbohydrate (CARB) diet [33]. Diets, and not the gut microbiota, were shown to affect not only the distribution of the gut microbiota communities (decrease in* Ruminococcaceae* and increase in* Rikenellaceae* with HF compared to CARB) but also the metabolome and proteome of the individual groups [33]. Although this study used two functional approaches to explore gut microbiota function, the numbers were very low (*n* = 3) which might have contributed to variation within the groups.

### 3.1. Conclusion from Animal Studies

In conclusion, the relationship of gut microbiota with diet and metabolic disorders has been studied in a variety of animal models. There is controversy as to whether these changes are attributable to the diet itself or are caused by the gut microbiota. Studies in germ-free mice suggest the gut microbiota are the critical player in inflammation, development of immunity, and host metabolic regulation. However, diet is also considered a confounding factor that determines a change in gut microbiota and obesity because the diversity of gut microbiota has not been found to be different between wild-type and certain genetic models of obese mice.

Discrepancies between and within studies could be attributed to the selection of animals (rats versus mice) and individual strains. A recent study by Walker et al. (2014) observed a distinct microbiome and metabolome in two strains of C57BL/6J and C57BL/6N mice [100]. Some differences in the metabolome might also be attributed to gender [96] and described above in addition to other methodological, host, and environmental differences. The exact mechanism of how these changes lead to an obesity phenotype is still not known. Large humans based interventional studies are therefore required to establish the true association between diet and gut microbiota and obesity.

## 4. Review of Human Studies Relating Gut Microbiota with Obesity

Evidence linking the gut microbiota with obesity in humans is thus far inconclusive and controversial. This may be partly due to marked interindividual variations in the gut microbiota and metabolic activity in humans with age, diet, use of antibiotics, genetics, and other environmental factors [101]. Apart from the interindividual variation in faecal microbiome and diversity, reanalysis of large datasets such as from the human microbiome project (HMP) and MetaHIT has shown interstudy variability which was far greater than the actual differences between the lean and obese phenotypes [102]. Refined statistical modelling therefore led to loss of some correlations previously found, such as between BMI and Firmicutes to* Bacteroides* ratio [102]. Bridging these gaps in analysis and accounting for these technical and clinical factors is therefore important to elucidate differences between normal and altered host microbiome and metagenome.

The first evidence showing higher Firmicutes and lower Bacteroidetes in obese versus lean adults before the onset of dietary intervention was presented by Ley et al. (2006) [5], followed by a number of studies reviewed in Table 6. Moreover, several gut microorganisms have been associated with obesity or leanness [44, 103] (Table 4). The type of gut microbiota and their exact phylogenetic level at which they exhibit differences are still under investigation. Evidence suggesting no phylum level differences between lean and obese gut microbiota [18, 65] may indicate that functionality of bacteria may play a more important role than particular bacterial groups.

The energy harvesting capability of the gut microbiota in obese subjects is thought to be set at a higher threshold than in the lean with or without differences in the relative abundance of the gut microbiota. Obese adults had higher individual and total SCFA than lean adults in the absence of any difference in the relative abundance of major gut bacterial phyla [29]. Moreover, no significant correlation of the gut microbiota with dietary factors in early [59] and later childhood [47] and a positive correlation with BMI SDS indicate that changes in the gut microbiota at these developmental stages may not depend on dietary factors.

On the other hand, evidence also suggests that diet plays an important role in altering the proportion of gut microbiota in individuals because the amount and type of bacteria change significantly with diet [64, 67]. This varies between individuals and may be due to the distinct microbiota colonisation during early life, altering the capacity for energy harvest from the diet. Composition and caloric content of the diet significantly alter the relative abundance of the gut microbiota [67]. An increased intake of resistant starch was associated with an increase in* Eubacterium rectale* (a butyrate producing bacteria) to ~10% and* Ruminococcus bromii* (an acetate producer) to ~17% compared with ~4% in volunteers consuming nonstarch polysaccharides [67]. These changes were reversed with weight loss diets along with a decrease in* Collinsella aerofaciens*, a member of Actinobacteria. This shows the substantial effect of diet on the gut microbiota and its energy harvesting capability [64, 67]. Similarly, SCFA production is affected by nutrient load and dietary carbohydrate available for fermentation. Weight loss diets usually have low carbohydrate and high protein content and reduce the population of butyrate producing* Roseburia* and* Eubacterium rectale* [66].

Long term changes in gut microbiota (such as lower counts of Bifidobacteria and higher* Bacteroides*) have been observed in children who were exposed to antibiotics in early childhood [104, 105]. Modulation of gut microbiota with antibiotics (e.g., norfloxacin and ampicillin) alters the expression of hepatic and intestinal genes involved in inflammation and metabolism thereby changing the hormonal, inflammatory, and metabolic milieu of the host [106]. These antibiotic induced changes may predispose children to overweight and obesity by promoting “obesogenic-bacterial-growth” (Table 5). The development of gut microbiota in infants and their tendency towards overweight and obesity in later childhood are linked to mother's prepregnancy BMI and gut microbiota with significantly lower numbers of faecal Bifidobacteria and* Bacteroides* and significantly higher* E. coli* and* Staph. aureus* in overweight and obese compared to normal weight pregnant women [64].

In addition to compositional differences between lean versus obese subjects [5], functional differences in the metabolome of the obese and lean phenotype may be more important. Calvani et al. (2010) in their preliminary study of 15 morbidly obese and 10 age matched controls found distinct gut microbial cometabolites in urine of obese versus lean participants, including lower levels of hippuric acid (benzoic acid derivative), trigonelline (niacin metabolite), and xanthine (purine metabolism) and higher levels of 2-hydroxybutyrate (metabolite of dietary protein) [49]. The metabolic or functional representation of gut microbiota might be proportional despite differences in the relative abundance of the gut microbiota. Disturbance of this equilibrium is a hallmark of the obese phenotype as suggested by Ferrer et al. (2013) in a comparative metagenomic and metatranscriptomic analysis of faecal samples from obese and lean adolescents [61]. Despite low compositional representation (up to 18%), up to 81% of the expressed proteins were contributed by Bacteroidetes [61]. Moreover, the obese metagenome had higher aerobic and anaerobic vitamin B12 and 1,2-propanediol metabolism genes than the lean which expressed genes related to vitamin B6 metabolism [61]. A recent study by Cottilard et al. (2013) has shown a reduced bacterial richness, reduced diversity, and higher dysmetabolism and low-grade inflammation in obese versus lean humans [71]. Although dietary intervention partially improved gene richness, reduced measures of adiposity such as waist circumference and fat mass, and reduced plasma cholesterol, it was less efficient in improving low-grade inflammation [71]. Furthermore, the tendency of the changes in gene clusters to return to the predietary restriction phase suggests that gut microbiota remain stable in individuals after the dietary stimulus is removed. Similarly, postgastric bypass surgery changes in gut microbiota and the expression of genes in obese subjects tend to reverse in the long term suggesting restructuring of the gut microbiota and a plateau of the response to changes in gut physiology [45]. Probiotics (such as* L. paracasei* strain F19) may beneficially affect short term energy homeostasis in weaning infants [107]. However, no differences in serum lipids, glucose, insulin, and anthropometry were seen in the F19 intervention group compared to placebo group when the same cohort of children were followed up at age of 8-9 years [108].

In this context, factors affecting colonisation of the gut microbiota in the newborn from before birth to early and late childhood might play an important role. However, the role of these factors in establishing a gut microbiota with tendency towards obesity or allergic disorders in later life is controversial. For example, higher numbers of Bifidobacteria and lower numbers of* Staphylococci* in breast fed children at age of 6 and 12 months had a negative correlation with overweight and obesity at 7 years [53]. Similarly, population based cohort study of a Danish National Birth Cohort did not show association of caesarean section with the development of overweight and obesity in more than 10,000 male children [51]. However, despite a larger cohort, the data was not adjusted for other confounding factors such as socioeconomic status and anthropometric and behavioural factors. In contrast, a recent Brazilian study following children born by caesarean section (*n* = 5914) at age of 4, 7, 15, and 23 years showed that although children born with caesarean section had ~50% higher prevalence ratio of obesity, this effect was lost when adjusted for socioeconomic, demographic, maternal, anthropometric, and behavioural factors [109].

### 4.1. Conclusions from Human Studies

Controversies exist as to whether or not obese and nonobese individuals host a particular type of bacterial phyla or enterotype and whether the response of the gut microbiota to diet differs. Correlation of BMI with* Bacteroides* in obese and nonobese subjects on different dietary regimens [65] is unclear as an inverse relationship has also been observed [18], adding to the complexity of the relationship of diet, gut microbiota, and obesity. The population of gut microorganisms in the human intestine is affected by a variety of factors from birth till adulthood, of which some are known and others are largely unknown. Additionally, large interindividual variations have been observed in all human studies suggesting host diet interaction at individual level.

## 5. Conclusion

The prevalence of obesity has increased in pandemic proportions in adults and children. Several factors have been identified to explain the aetiology and pathogenesis of obesity including diet, lifestyle, environmental factors, and host genetic factors. However, none of these fully explain the aetiology of obesity and the search for possible causes continues. The gut microbiota have been advocated as one factor affecting host energy homeostasis through several putative mechanisms investigated in mice models and human studies. However, several studies have suggested a profound effect of diet on the gut microbiota which modify host metabolism towards a lean or obese phenotype.

The evidence linking gut microbiota to the increasing epidemic of obesity is too contradictory and inconclusive to prove a “cause or effect” relationship. This may be due to differences in methodology, study design, control of diet, genetic propensity of individuals to obesity, and other lifestyle factors. Moreover, faecal samples are the usual source of gut microbiota which may not represent the true picture of the colonic microbial population. Access to the full length of the gut is restricted for medical or ethical reasons. In addition, differences between animals and human beings including intestinal microbiota, metabolic rate, and length of intestine, caecal fermentation, coprophagy, and genetic variability limit the extrapolation of results from animal studies.

## Figures and Tables

**Table 1:** Suggested mechanisms for the role of gut microbiota in the aetiology of obesity.

	Proposed mechanism	Mediators	Source of mediators	Target tissues/organs	Local/systemic effects
Metabolic	Increased production of short chain fatty acids [1]	Bacterial glycosyl hydrolases	Colon, distal ileum, and rectum	Colonic enterocytes	↑ energy harvest Energy for colonocytes Alteration in cholesterol metabolism
	Muscle fatty acid oxidation [1]	↓ AMP kinase	Small intestine	Muscle, liver	↓ muscle fatty acid oxidation
	Bile acid circulation [19]	Secondary bile acid production	Colon	Colon	Reverse cholesterol transport
	Expression of liver ChREBP/SREBP-1 [1]	↑ glucose absorption	Liver	Liver	↑ hepatic lipogenesis

Inflammatory	Chronic low-grade inflammation [9]	LPS, NF-kappaB, and TNF-*α* mRNA	Colon, ileum	Endothelium, hypothalamus?	Metabolic endotoxemia and hyperphagia
	↑ endocannabinoid (eCB) system tone [10, 20]	Bacterial LPS	Ileum, colon	Stomach, small and large intestine	↑ gut permeability and ↓ apelin and APJ mRNA expression

Hormonal	Suppression of Fiaf [1]	Colonic L-cells	Colon	Adipose tissue	↑ lipolysis, ↓ muscle fatty acids oxidation
	↑ PYY [21]	Satiety centre	Ileum, colon	Hypothalamus	↓ appetite, ↓ gastric motility, and ↓ gut emptying
	Expression of G protein coupled receptors 41 and 43 (GPR41 and GPR43) [22]	SCFA (acting as a ligand)	Colon, distal ileum, and rectum	Liver, brain	↑ peptide YY (PYY), ↑ *de novo* hepatic lipogenesis

AMP: adenosine monophosphate, ChREBP: carbohydrate response element binding protein, SREBP-1: sterol response element binding protein-1, PYY: peptide YY, LPS: lipopolysaccharide, NF-kappaB: nuclear factor-kappaB, TNF-*α*: tumour necrosis factor alpha, mRNA: messenger RNA, GPR41 and GPR43: G protein coupled receptors 41 and 43, SCFA: short chain fatty acid, and eCB: endocannabinoid.

**Table 2:** Studies looking at differences in SCFA in faecal or caecal samples in obese versus lean phenotypes in animal and human studies.

Reference	Technique used	SCFA differences	Gut microbiota differences
Turnbaugh et al. 2006 [4]	GC-MS, pyrosequencing	↑ caecal acetate and ↑ butyrate in obese ob/ob mice compared to lean	↑ Firmicutes and lower Bacteroidetes in obese than lean mice. No differences in genera level diversity
Zhang et al. 2009 [23]	GC, qPCR, and pyrosequencing	↑ acetate in obese compared to lean and gastric bypass group	↑ *M. smithii* and Prevotellaceae in obese compared to lean and gastric bypass
Schwiertz et al. 2010 [18]	GC and qPCR with SYBR Green	↑ total SCFA and propionate (conc. & %) in obese compared to lean	↑ *Bacteroides* and ↓ Firmicutes, ↓ *Ruminococcus flavefaciens*, ↓ *Bifidobacterium*, and ↓ *Methanobrevibacter* in obese compared to lean
Payne et al. 2011 [24]	qPCR, TGGE, and HPLC	↑ butyrate, propionate, and isobutyrate in obese compared to lean ↑ lactate and valerate in lean compared to obese No difference in acetate and total SCFA	No difference in Firmicutes and Bacteroidetes, Firmicutes/*Bacteroides* ratio, Bifidobacteria, Enterobacteriaceae, and sulphate reducing bacteria between lean and obese children ↑ *Roseburia/E. rectale* in obese Highly variable banding pattern on TGGE for both obese and healthy

Yang et al. 2013 [25]	GC	↑ ratio of molar propionate: total SCFA and ↓ acetate : SCFA ratio in obese versus lean	Not measured
Teixeira et al. 2013 [26]	GC	↑ acetate, propionate, and butyrate in obese versus lean women SCFA correlated with body fat, blood pressure, waist circumference, insulin, and HOMA index	Not studied
Belobrajdic et al. 2012 [27]	GC	Increase in total SCFA pool and stool energy irrespective of obese or lean phenotype (obesity prone or obesity resistant) in response to 0, 4, 12, and 16% resistant starch diet for 4 weeks	Not studied
Rahat-Rozenbloom et al. 2014 [28]	GC	↑ total SCFA, acetate, and butyrate in obese compared to lean No differences in isobutyrate, isovalerate, and valerate	↑ Firmicutes : Bacteroidetes ratio in obese. Firmicutes correlated with SCFA in obese
Fernandes et al. 2014 [29]	GC, qPCR	Significantly ↑ propionate and valerate Marginally ↑ acetate and butyrate	*Escherichia Coli* higher in lean than obese No difference in *Bacteroides/Prevotella, Clostridium coccoides and C. leptum* group, Bifidobacteria, and total bacteria, F/B ratio
Li et al. 2013 [30]	GC	Higher SCFA in obese than lean	↑ Firmicutes and lower Bacteroidetes in obese

GC: gas chromatography, GC-MS: gas chromatography-mass spectrometry, SPME-GCMS: solid phase microextraction-gas chromatography mass spectrometry, v1-v2: variable regions 1 and 2, HPLC: high performance liquid chromatography, TGGE: temperature gradient gel electrophoresis, CHO: carbohydrate, EU: European Union, qPCR: quantitative polymerase chain reaction, and F/B ratio: Firmicutes to Bacteroidetes ratio.

**Table 3:** Evidence from animal studies about the role of gut microbiota in obesity.

Reference	Study model	Aim of the study	Study design and outcomes measures	Results	Conclusion
*Studies suggesting association of gut microbiota with obesity*					

Fleissner et al. 2010 [14]	Male adult C3H GF and CV mice	Influence of different diets on the body composition of GF and CV mice	*Ad libitum* intake of low fat (LF), high fat (HF), and commercial Western diet (WD) for GF and CV mice. Real-time PCR, FISH, and *fiaf*/angplt4 in gut and blood	GF mice gained more weight and body fat and had less energy expenditure than CV mice on HF. Higher Firmicutes (especially Erysipellotrichacae) and lower *Bacteroides* in CV mice on HF and WD. Intestinal *Fiaf* increased in GF mice but no change in plasma fiaf levels as compared to CV mice	GF mice are not protected from diet induced obesity. Diet affects gut microbiota composition and *fiaf* does not play a role in fat storage mediated by gut microbiota

Šefčíková et al. 2010 [31]	8–10 pups per nest, Sprague-Dawley rats, from day 21 to day 40	Effect of normal and overnutrition on the development of gut microbiota, intestinal alkaline phosphatase, and occurrence of obesity	Standard laboratory diet for control group and additional milk based liquid diet for study group. Bacterial enumeration via FISH, alkaline phosphatase activity via immunocytochemistry	Obese rats gained more energy (25%) and higher body fat (27%) than lean rats. Alkaline phosphatase increased in obese rats. Lactobacilli increased while *Bacteroides* decreased in obese rats significantly	This study may provide a baseline for further insight into the ways of involvement in programming of a sustained intake and digestion

Ding et al. 2010 [32]	GF/CONV mice and NF-*κ*B knockin mice (GF/CONV)	Hypothesis: intestinal inflammation is promoted by the interaction of gut bacteria and high fat diet, contributing to the progression of insulin resistance and obesity	High and low fat diets for 2, 6, or 16 weeks. GF mice fed with diet after exposure to faecal slurries of CONV mice. Blood glucose and ELISA for insulin. TNF-*α* mRNA expression by qPCR. Expression of NFkB mice by fluorescent light microscopy	CONV mice gained more weight than GF. Increased expression of TNF-*α* mRNA and NF-*κ*B in CONV HF diet mice. TNF-*α* changes precede weight changes. Enhanced NF-*κ*B in GF NF-*κ*B mice on feeding CONV NFkB faecal slurry	HF diet and enteric bacteria interact to promote inflammation and insulin resistance prior to the development of weight gain, adiposity, and insulin resistance

Turnbaugh et al. 2008 [17]	8-9-week-old GF/CONV mice	To study the interrelationship between diet, energy balance, and gut microbiota using mouse model of obesity	Conventionalisation of GF mice with HF Western diet followed by introduction of Western or CHO diet in CONV mice. CARB-reduced or FAT-reduced diets in another subset. qPCR, DEXA scan, and weight measurements done	Western diet-associated caecal community had a significantly higher relative abundance of the Firmicutes (specifically Mollicutes) and lower Bacteroidetes. Mice on the Western diet gained more weight than mice maintained on the CHO diet and had significantly more epididymal fat. Mice on CARB-R and FAT-R diet consumed fewer calories, gained less weight, and had less fat	There is restructuring of gut microbiota with Western diet, specifically reduction of *Bacteroides* and surge in Mollicutes class of Firmicutes with increased capacity to harvest energy from diet

Daniel et al. 2014 [33]	Male C57BL/6NCrl mice (*n* = 6, per group)	To investigate changes in function and activity of the gut ecosystem in response to dietary change	LC-MS/MS for metaproteome, FT-ICR-MS for metabolome, Miseq illumina pyrosequencing. Intervention with high fat (HF) and control (carbohydrate) diet for 12 weeks	HF diet did not affect caecal taxa richness. Bacterial communities clustered according to diet. Significantly ↓ *Ruminococcaceae* (Firmicutes) and ↑ *Rikenellaceae* (phylum Bacteroidetes)*, Lactobacilli, *and *Erysipelotrichiales *in HF fed versus carbohydrate fed diet. 19 OTUs affected by HF diet. Carbohydrate and HF group had distinct proteome and metabolome	High fat diet affects gut microbial ecology both in terms of composition and function

Cani et al. 2007 [9]	C57bl6/J mice and CD14−/− mutant strain	To evaluate the influence of gut microbiota on the development of metabolic endotoxemia	Metabolic, inflammatory, and microbiological differences (by FISH) between high fat fed obese or rodent lean chow-fed mice	High fat feeding and obesity decimate intestinal microbiota– *Bacteroides-*mouse intestinal bacteria, *Bifidobacterium,* and *Eubacterium rectale-Clostridium coccoides* groups all significantly ↓ compared to in control animals	High fat diet induces changes in gut microbiota that leads to elevated plasma LPS leading to metabolic endotoxemia, by altering the gut barrier function

Cani et al. 2008 [34]	C57bl6/J *ob/ob* mice	Manipulating gut microorganisms through antibiotics to demonstrate whether changes in gut microbiota control the occurrence of metabolic syndromes	Caecal microbiota of mice under high fat low fibre diet and antibiotics. qPCR and DGGE	Antibiotic reduced LPS caecal content and metabolic endotoxemia in both *ob/ob* and high fat groups. High fat diet ↑ intestinal permeability and LPS uptake leading to metabolic endotoxemia. Absence of CD14 mimicked the metabolic and inflammatory effects of antibiotics	High fat diet modifies gut microbiota which induce inflammation and metabolic endotoxemia. Antibiotics can reverse these changes

Murphy et al. 2010 [16]	HF fed wild-type mice and leptin deficient *ob/ob* mice (*n* = 8 per group)	To investigate the effect of high fat diet and genetically determined obesity for changes in gut microbiota and energy harvesting capability over time	GC, metagenomic pyrosequencing high fat or normal chow diet fed to *ob/ob* mice and wild-type mice for 8 weeks	↑ in Firmicutes and Bacteroidetes in HF fed and obese mice but not in lean. Changes in microbiota not associated with markers of energy harvest. Initial increase in caecal SCFA (acetate) and ↓ in stool energy with HF diet did not remain significant over time	Changes in bacterial phyla are a function of high fat diet and are not related to the markers of energy harvest

de Wit et al. 2012 [35]	Male C57BL/6J mice	To study the effect of dietary fat type (polyunsaturated and saturated fatty acids ratio) on the development of obesity	Phylogenetic microarray (MITChip) analysis, bomb calorimetry, measurement of triglycerides, and plasma insulin	HF diet with high saturated fatty acids (palm oil) induced ↑ weight gain and liver TG compared to HF diet with olive oil and safflower oil. HF diet with palm oil ↓ microbial diversity and ↑ Firmicutes (Bacilli, *Clostridium clusters* XI, XVII, and XVIII) Bacteroidetes ratio. Upregulation of 69 lipid metabolism genes in distal small intestine and ↑ fat in stool	Type of dietary fat influences the weight gain and hepatic lipid metabolism

Faith et al. 2011 [36]	Male C57BL/6J mice (*n* = 10 per group)	Changes in 10 model gut communities species' abundance and microbial genes with changes in peculiar diet	Shotgun sequencing of faecal DNA diets used for each community: casein (for protein), corn oil (for fat), starch (for polysaccharides), and sucrose (for simple sugars)	61% variance in abundance of the community members was explained by diet particularly casein. Absolute abundance of *E. rectale, Desulfovibrio piger,* and *M. formatexigens* ↓ by 25–50% while *Bacteroides caccae* ↑ with increase in casein, although the total community biomass ↑	Host diet explains configuration of gut microbiota both for refined diets and complex polysaccharides

Hildebrandt et al. 2009 [37]	RELM-*β* knockout female mice and wild-type mice	To assess the influence of host phenotype, genotype, immune function, and diet on gut microbiota	16S rDNA 454 FLX pyrosequencing, metagenomic sequencing	Switching to high fat diet caused ↓ Bacteroidetes and ↑ Firmicutes and Proteobacteriain both wild-type and RELM-*β* knockout mice irrespective of the genotype. Genetic makeup only modestly influenced the gut microbiome composition. Changes in gene content with HF diet	Diet determines the gut microbiota composition

Huang et al. 2013 [38]	Adult male C57BL/6	To assess the relationship of diet content and source on gut microbiota and adiposity	16S rRNA analysis, terminal restriction fragment length polymorphism and V3-V4 sequence tag analysis via next generation sequencing. Mesenteric fat and gonadal fat tissue analysis. Milk, lard, or safflower based diets for 4 weeks	↑ weight gain and caloric intake with HF compared to low fat diet. Milk based and PUFA based diets animals had ↑ adipose tissue inflammation than lard based or low fat diet. Milk based and PUFA diet had significantly ↑ Proteobacteria and ↓ *Tenericutes. *PUFA based fed animals had ↑ expression of adipose tissue inflammation genes (MCP1, CD192, and resistin)	Dietary fat components reshape gut microbiota and alter adiposity and inflammatory status of the host

Jakobsdottir et al. 2013 [39]	Male Wister rats	To investigate the effect of dietary fibre on metabolic risk markers in low and high fat diets at 2, 4, and 6 weeks	Gas liquid chromatography, liver fat content, cholesterol and triglycerides analysis, and terminal fragment length polymorphism. Diets supplemented with guar gum or a mixture	↓ in weight gain, liver fat, cholesterol, and triglycerides with fibre. Change in formation of SCFA. ↓ in serum SCFA with HF diet followed by recovery after 4 weeks. Succinic acid ↑ with HF consumption. Dietary fibre ↓ this effect and also ↓ inflammation. *Bacteroides* were ↑ with guar gum and *Akkermansia* was ↑ with fibre-free diet	HF diet ↑ metabolic risk factors which are partly reversed by high fibre diet

*Studies suggesting effect of diet on changes in gut microbiota and resultant obesity*					

de la Serre et al. 2010 [15]	Male Sprague-Dawley rats	To evaluate whether changes in gut bacteria and gut epithelial function are diet or obese associated	Intestinal permeability, intestinal Alk-Pase, plasma LPS, tissue myeloperoxidase (MPO) activity, immunochemical localization of TLR4/MD2 complex, and Occludin. Sequence analysis of the microbial 16S rRNA gene	Appearance of two distinct groups; diet induced obesity prone (DIO-P) and diet induced obesity resistant (DIO-R) groups. DIO-P rats had ↑ features of adiposity, ↑ MPO activity, ↑ TLR4 MD2 immunoreactivity and ↑ plasma LPS levels, ↑ gut permeability, immunoreactivity of Occludin, and ↓ alkaline phosphatase levels than LF and DIO-R group. HF diet was associated with ↑ Clostridiales regardless of propensity for obesity. A marked difference in Enterobacteriales in DIO-P animals compared with either DIO-R or LF fed animals	Changes in gut bacteria are independent of obese status. Gut inflammation marked by increased LPS may be a triggering mechanism for hyperphagia and obesity

Bäckhed et al. 2004 [1]	Adult germ-free (GF) C57BL/6 mice	To evaluate the effect of gut microbiota on the host energy metabolism using animal model	Conventionalisation of GF mice with murine gut microbiota or *B. thetaiotaomicron*, intestinal *fiaf*, liver metabolism, total body fat, LPL activity in adipose tissue, and faecal microbiota composition by qPCR	Conventionalized GF mice showed 57% ↑ in body fat, increased energy expenditure, ↓ intestinal *fiaf*, increased LPL activity, and ↑ expression of ChREBP and SREBP-1 in liver. Firmicutes to *Bacteroides* ratio similar in GF and CONV	Gut microbiota alter host energy storage by affecting *fiaf *and LPL activity

Bäckhed et al. 2007 [13]	Adult GF C57BL/6 mice (*n* = 5) and CONV mice (*n* = 5)	To assess whether GF mice are protected against obesity on high fat Western diet	Dietary intervention with low fat followed by high fat Western diet for 8 weeks	CONV mice gained ↑ weight on HF diet while conventionalised GF mice did not. Stool energy was similar to the LF fed GF mice. Persistent ↑ TG in HF fed GF mice. GF mice had ↑ Acc-p, AMPK-P, and Cpt-1 activity. GF mice had ↓ hepatic glycogen and glycogen-synthase activity. ↑ *fiaf* in HF fed GF mice	GF mice are protected against diet induced obesity by two mechanisms: (1) increased phosphorylated AMPK and (2) increased *fiaf*

Vijay-Kumar et al. 2010 [40]	TLR5 knockout mice (T5KO), wild-type mice (WT)	To show that mice deficient in TLR-5 exhibit hyperphagia, which is a principal factor in the development of obesity and metabolic syndrome	Broad spectrum antibiotics. Pyrosequencing of 16S rRNA genes in the caecum. Transplantation of TLR5-KO mice microbiota into WT germ-free hosts	Antibiotic treatment ↓ the bacterial load by 90%, correction of metabolic syndrome similar to the wild-type mice. Relative abundance of bacterial phyla was similar in both, with 54% Firmicutes, 39.8% *Bacteroides*. 116 phyla observed to be enriched or ↓ in TLR5-KO relative to WT mice. Microbiota of WT mice transplanted to the TLR5-KO mice resulted in all features of metabolic syndrome in the TLR5-KO group	Loss of TLR-5 results in metabolic syndrome and alteration in gut microbiota

Ley et al. 2005 [3]	Leptin deficient C57BL/6J *ob/ob* mice, lean *ob/+,* and *+/+* mice (*n* = 19)	To study differences in bacterial diversity between obese genetic model of obesity and its relationship with kinship	16S rRNA gene amplification of caecal bacteria followed by analysis using PHRED and PHRAP software. All mice fed the same polysaccharide rich chow	*ob/ob* mice consumed 42% more chow and gained significantly ↑ weight. Mothers and offspring shared bacterial community. Obese *ob/ob* mice had 50% reduction in Bacteroidetes and a proportional ↑ in Firmicutes as compared to lean regardless of the kinship and gender	Obesity is associated with altered bacterial ecology. This however needs to be correlated with the metabolic attributes of gut microbial diversity

Turnbaugh et al. 2006 [4]	Leptin deficient C57BL/6J *ob/ob* mice (*n* = 13) and lean ob/+ and +/+ mice (*n* = 10)	Whether gut microbial gene content correlates with characteristic distal gut microbiome of leptin deficient ob/ob mice and their lean counterparts	1S rRNA whole genome shotgun metagenomics, GC-MS for SCFA analysis, bomb calorimetry, gut microbiota transplantation, and DEXA	Firmicutes-enriched obese microbiome clustered together while lean phenotype with ↓ Firmicutes to Bacteroidetes ratio clustered together. Obese microbiome rich in enzymes for breakdown of dietary polysaccharides particularly glycoside hydrolases. *ob/ob* had ↑ acetate and butyrate and significantly ↓ stool energy	Obese microbiome is associated with increased energy harvest

Caesar et al. 2010 [12]	Swiss-Webster mice (GF, CONV, and *E. coli* monocolonised mice)	Whether gut microbiota especially LPS promote inflammation in white adipose tissue (WAT) and impair glucose metabolism	DEXA, insulin, and glucose tolerance. Macrophage isolation, immunohistochemistry, and flow cytometry and immunoblot in WAT, LPS analysis, and RT-qPCR	Monocolonisation of GF mice with *E. coli* W3110 or isogenic strain MLK1067 with low immunogenic LPS had impaired glucose tolerance. However, only GF mice with *E. coli* W3110, and not MLK1067, showed ↑ proinflammatory macrophage infiltration in WAT	Macrophage accumulation is microbiota dependent but impaired glucose tolerance is not

Caricilli et al. 2011 [41]	TLR2 knockout mice (TLR2−/−) and wild-type mice (*n* = 8 per group)	Influence of gut microbiota on metabolic parameters, glucose intolerance, insulin sensitivity, and insulin signalling in TLR2 knockout mice	454 pyrosequencing	↑ Firmicutes (47.92% versus 13.95%), Bacteroidetes (47.92% versus 42.63%), and ↓ Proteobacteria (1.04% versus 39.53%) in TLR2−/−. ↑ LPS absorption, insulin resistance, impaired insulin signalling, and glucose intolerance in TLR2−/− compared to controls	Alteration in gut microbiota in non-germ-free conditions links genotype to phenotype

Everard et al. 2013 [42]	C57BL/6 mice (genetically obese, HF fed, and type-2 diabetic)	To ascertain the role of *Akkermansia muciniphila* in obesity and type-2 diabetes	Real-time qPCR, MITChip analysis, LTO-Orbitrap mass spectrometer, and ELISA for insulin and faecal IgA	*Akkermansia muciniphila* ↓ obesity and type-2 diabetes which was normalised by oligofructose. Administration of *A. muciniphila* reversed markers of metabolic disorders. These effects needed viable *A. muciniphila*	This microorganism could be used as part of a potential strategy for the treatment of obesity

Fei and Zhao 2013 [43]	C57BL/6J GF mice	Endotoxin producing *Enterobacter cloacae* B29 isolated from obese human gut could induce obesity and insulin resistance in GF mice	16S rRNA gene sequencing for bacteria and limulus amebocyte lysate test for endotoxin measurement	Monocolonisation of GF mice with *E. cloacae* induced obesity and insulin resistance on HF diet while GF control mice only on HF diet did not. *Enterobacter-*colonised GF obese mice had ↑ plasma endotoxin levels and inflammatory markers	Gut microbiota-produced endotoxin may be causatively related to obesity in human hosts

Geurts et al. 2011 [20]	Leptin resistant *db/db* mice	To investigate the gut microbiota composition in obese and diabetic leptin resistant mice versus lean mice	Combined pyrosequencing and phylogenetic microarray analysis of 16S rRNA gene	↑ Firmicutes, Proteobacteria, and Fibrobacteres phyla in *db/db* mice compared to lean mice*. Odoribacter, Prevotella*, and *Rikenella* were exclusively present in *db/db* mice while *Enterorhabdus* was identified exclusively in lean mice. *db/db* mice had ↑ tone of eCB and ↑ apelin and APJ mRNA levels	Gut microbiota vary with genotype and play a significant role in the regulation of eCB and apelin/APJ mRNA system

GF: germ-free mice, CV: conventionally raised germ-free mice, HF: high fat diet, LF: low fat diet, WD: Western diet, PCR: polymerase chain reaction, FISH: florescent *in situ* hybridization, fiaf/angptl4: fasting induced adipocyte factor/angiopoietin-like-protein factor-4, NF-*κ*B: nuclear factor-kappaB, CHO: carbohydrate, CARB-R: carbohydrate-reduced diet, FAT-R: FAT-reduced, DEXA or DXA: dual energy X-ray absorptiometry, FT-ICR-MS: Fourier-transform ion cyclotron resonance mass spectrometry, OTUs: operational taxonomic units, LPS: lipopolysaccharide, DGGE: denaturing gradient gel electrophoresis, GC: gas chromatography, SCFA: short chain fatty acids, RELM-*β*: resistin-like molecule-*β*, PUFA: polyunsaturated fatty acids, MCP1: monocyte chemoattractant protein 1, Alk-Pase: alkaline phosphatase, TLR4/MD2: Toll-Like Receptor 4/mitogen detector-2, ChREBP: carbohydrate response element binding protein, SERBP-1: sterol response element binding protein-1, TG: triglycerides, Cpt-1: carnitine palmitoyltransferase-1, AMPK: adenosine monophosphate kinase-1, Acc-p: acetyl CoA carboxylase (phosphorylated), WT: wild-type, GC-MS: gas chromatography-mass spectrometry, and eCB: endocannabinoid receptor system.

**Table 4:** Association of gut microbial species/genera with obesity or leanness in human studies.

Bacteria	Association^*∗*^ with obesity	Group	Level	Other associations	Reference
*Lactobacillus reuteri*	+ve	Firmicutes	Species	—	[44, 45]
*Clostridium cluster* XIVa	+ve	Firmicutes	Group	Anti-inflammatory	[46]
*E. coli*	+ve	Proteobacteria	Species	Nonalcoholic steatohepatitis (NASH)	[46]
*Staphylococcus *spp.	+ve	Firmicutes	Genus	Energy intake	[47]
*Bacteroides*	−ve/+ve	Bacteroidetes	Genus	Controversial	[5]
*Akkermansia muciniphila*	−ve	Verrucomicrobia	Species	Mucus degradation	[42]
*Methanobrevibacter smithii*	−ve	Archaea	Species	Increase in anorexia	[48]
*Clostridium cluster *IV*; F. prausnitzii*	−ve	Firmicutes	Species	Anti-inflammatory	[49]
Bifidobacteria	−ve	Actinobacteria	Genus	−ve association with allergy	[44]

^*∗*^Associations based on correlation or regression analysis or statistically significant differences between the lean and obese. +ve: positive association, −ve: negative association, and +ve/−ve: controversial.

**Table 5:** Population based studies to investigate the risk of obesity and overweight in children who were given antibiotics for treatment of infections in early infancy.

Study reference	Design and population	Age group	Tools	Primary outcome	Factors considered	Findings
ISAAC study (International Study of Asthma and Allergies in Childhood) [50]	*n* = 74,946 cross-sectional	5–8 years	Questionnaires/interviews, measurements	Antibiotics use in first 12 months of life	Ht., Wt., BMI, age, gender, antibiotics, paracetamol, breast-feeding, maternal smoking, gross national income, and asthma	Association of antibiotics use and BMI in boys (+0.107 kg/m^2^ *p* < 0.0001), not in girls even after adjustment for the other variables

DNBC study (Danish National Birth Cohort) [51]	*n* = 28,354	Up to 7 years	Questionnaires/telephonic interviews based	Antibiotics use in <6 months of life	Socioeconomic status, maternal age and smoking, gestational weight gain, parity, delivery mode, breast-feeding, paternal BMI, birth weight, and age at 7-year follow-up	Increased risk of overweight in children born to normal weight mothers (adjusted OR: 1.54, 95% CI: 1.09–2.17) and especially in boys when adjusted for maternal age, smoking, SE status, birth weight, and breast-feeding

ALSPAC study (Avon Longitudinal Study of Parents and Children) [52]	*n* = 11,532 longitudinal	7 years	Questionnaires based, hospital records, and objective measurements	Antibiotic exposure at <6 months, 6–14 months, and 15–23 months and BMI at 6 weeks, 10 months, 20 months, 38 months, and 7 years	Maternal parity, social class, education, parental BMI, parental smoking, breast-feeding, lifestyle, and dietary patterns	Increased risk of overweight at 38 months (OR 1.22, *p* = 0.029) but not at 7 years in children exposed to antibiotics <6 months

**Table 6:** Evidence from human studies about the role of gut microbiota in obesity.

Reference	Study model	Aim of the study	Study design and outcomes measures	Results	Conclusion
*Studies suggesting association of gut microbiota with obesity*					

Kalliomäki et al. 2008 [53]	Children, 25 obese and 24 normal weight at 7 years of age	To evaluate whether differences in gut microbiota at an early age precede the development of obesity	Subjects examined at 3, 6, 12, and 24 months and 7 years. Gut microbiota composition at age of 6 and 12 months by FISH, FISH with flow cytometry, and qPCR	↑ Bifidobacteria numbers and ↓ *S. aureus *at 6 and 12 months of age in children remaining normal wt. ↑ *Bacteroides* in obese and overweight children during 6 and 12 months versus normal wt. children	↑ numbers of Bifidobacteria and ↓ numbers of *S. aureus* in infancy may provide protection against overweight and obesity development

Zhang et al. 2009 [23]	3 Obese (OB), 3 normal wt. (NW), and 3 postgastric bypass (GB) patients	To compare the gut microbial community of normal wt., morbidly obese, and postgastric bypass surgery patients	DNA pyrosequencing and amplification by real-time PCR	GB group had a marked increase in Gammaproteobacteria, Enterobacteriaceae, and Fusobacteriaceae and fewer Clostridia. Prevotellaceae (H_2_ producing) enriched in the OB group compared with the NW group. Methanogenic Archaea (H_2_ consuming bacteria of the group Archaea) were found ↑ in obese group	Suggests an association between methanogenic Archaea and obesity

Nadal et al. 2009 [54]	39 obese adolescents	Effect of weight loss intervention on the faecal gut microbial composition and immunoglobulin coating bacteria and its relationship to wt. loss	Restricted calories diet and ↑ physical activity for 10 weeks. BMI, BMI *z*-scores before/after intervention. FISH and fluorescent-labelled F(ab′)2 anti-human IgA, IgG, and IgM	*Clostridium histolyticum, Eubacterium rectale-Clostridium coccoides* groups' ↓ count with wt. loss. *Bacteroides Prevotella* ↑ and total faecal energy decreased upon weight loss of >4 kg. IgA coating bacteria ↓ with weight loss of >6 kg	Changes in adolescents' body wt. are linked to specific gut microbiota and an associated IgA response in obesity after lifestyle interventions

Tiihonen et al. 2010 [55]	40 obese and nonobese adults	To compare obese and lean individuals' gut bacterial and immunological biomarkers with blood glucose, lipids, satiety related hormones, and inflammatory markers	Interview for dietary fibre, anthropometry, faecal sample for microbiota diversity using PCR, and inflammatory markers. Blood biochemistry for hormones and inflammatory markers	IL6, CRP, insulin, TG, and leptin ↑ in obese. BCFA and phenolics ↑ in obese faecal samples indicate ↑ bacterial fermentation due to protein rather than carbohydrates. Waist circumference and Bacteroides were −vely correlated while they were +vely correlated with IL-6	↑ phenolics and lactic acid in intestine of obese subjects most probably have an effect on the physiology of systemic inflammatory condition

Larsen et al. 2010 [56]	36 adults; diabetic (*n* = 18) and nondiabetic controls (*n* = 18)	To assess the differences between gut microbiota of diabetic and nondiabetic persons	Bacterial composition of faecal samples by real-time PCR and by tag-encoded amplicon pyrosequencing of V4 region of 16S rRNA gene	*Bacteroides*, Proteobacteria, and Lactobacilli ↑ in diabetics and Firmicutes (clostridium group) were ↑ in nondiabetics. Ratio of *Bacteroides Prevotella* group to *C. coccoides-E. rectale *group and Β-Proteobacteria +vely correlated with glucose and *E. rectale *group −vely correlated with BMI	Reverse F : B ratio in diabetic patients indicates a different bacterial composition in this group. ↑ number of Gram negative bacteria may explain the chronic low-grade inflammation in diabetic patients

Santacruz et al. 2009 [57]	18 male and 18 female overweight and obese adolescents	To evaluate the influence of weight loss intervention on the gut microbiota and body wt. of overweight adolescents	Energy restricted diet and ↑ physical activity to all participants. Anthropometric measurements, food diaries, and faecal sample for qPCR	In overall groups and in high wt. loss group (>4 kg); ↑ in *Bacteroides fragilis, Lactobacillus* group and ↓ in *C. coccoides, Bifidobacterium longum,* and *Bifidobacterium adolescentis*. In high versus low wt. loss groups (<2 kg): total bacteria, *B. fragilis* group and *Clostridium leptum* group, and *Bifidobacterium catenulatum* group counts significantly ↑ while levels of *C. coccoides group*, *Lactobacillus* group, *Bifidobacterium*, *Bifidobacterium breve*, and *Bifidobacterium bifidum* significantly ↓	Correlation of gut microbiota with body wt. may be sensitive to the lifestyle intervention such as wt. loss to a different extent depending on the composition of gut microbiota of an individual

Armougom et al. 2009 [48]	Obese (*n* = 20), normal weight (*n* = 20), and anorexia nervosa (*n* = 9)	To determine the role of *Methanobrevibacter smithii* and *Lactobacilli* in patients with abnormal weights using real-time PCR	Real-time PCR	↓ in the *Bacteroidetes* community and ↑ *Lactobacillus species* in obese patients compared to in lean controls or anorexic patients. *M. smithii* much ↑ in anorexic patients compared to in the lean population	*Lactobacilli* used as probiotics may be linked to obesity*. M. smithii* in anorexia nervosa patients may represent an adaptive response to the disease

Collado et al. 2008 [58]	Overweight and obese mothers (*n* = 16) with their infants and nonobese mothers (*n* = 26) with their infants	To evaluate the faecal microbiota of infant born to overweight and normal wt. mothers and to find out their relationship with the weight and weight gain of mothers during pregnancy	Faecal sampling of infants, weight of mothers before and during pregnancy. Real-time PCR and FISH with flow cytometry for bacterial composition	*Bacteroides* and *S. aureus *↑ in infants of overweight mothers. Higher weights and maternal BMI related to ↑ concentrations of* Bacteroides*, *Clostridium*, and *Staphylococcus* and ↓ concentrations of the Bifidobacterium group. ↓ counts of *Akkermansia muciniphila*, *Staphylococcus*, and *Clostridium difficile* groups and ↑ number of Bifidobacteria in infants of normal wt. mothers and those with normal pregnancy wt. gains	Lower Bifidobacteria and higher *Staph. aureus* associated with obesity in children. BMI, weight, and wt. gain of mothers before and during pregnancy affect the gut microbiota composition in infants

Ley et al. 2006 [5]	12 obese human adults, followed up over a period of 1 year	To investigate the relative abundance of gut microbiota in obese people versus lean individuals	16S rRNA gene sequence library of gut microbiota in obese subjects on wt. reduction diets (low carbohydrate or low fat, *n* = 12)	Gut bacteria are remarkably constant in individuals. Relative proportion of Bacteroidetes ↑ compared with Firmicutes and correlated with percentage of wt. loss	The gut in obesity exerts ecological pressure promoting a higher relative abundance of Firmicutes

Ajslev et al. 2011 [51]	28 354 mother-child dyads, age of 7 years	To assess the influence of delivery mode, maternal prepregnancy BMI, and child's early exposure to antibiotics on the child's risk of overweight	Maternal prepregnancy BMI, delivery mode, and antibiotic administration in infancy. Children followed up at 7 years of age	No significant association of delivery mode with overweight. ↑ risk of overweight and obesity in children, born to normal wt. mothers given antibiotics in first 6 months of life and ↓ risk in children born to overweight mothers	Antibiotics use in early infancy and prepregnancy weight of mother affect tendency of child to become overweight and obese

Bergström et al. 2014 [59]	Healthy Danish infants (*n* = 330) at 9, 18, and 36 months of age	Characterisation of gut microbiota of infants at different ages	qPCR, DXA, and bioelectrical impedance analysis for body composition, barcoded food diary for 7 days for dietary analysis	At 9 months: higher *Lactobacilli*, Bifidobacteria, and *Enterobacteria*. At 18 months: Firmicutes (particularly *C. leptum, E. halii, and Roseburia*) and Bacteroidetes ↑ while Bifidobacteria*, Lactobacilli*, and *Enterobacteria ↓ *except *B. adolescentis. At 36 months: *↑ Firmicutes, Bacteroidetes, and small fraction of Actinobacteria, Proteobacteria, and Verrucomicrobia.↑ in BMI between 9 and 18 months was associated with ↑ Firmicutes	Significant differences occur between 9 and 18 months, and changes at 36 months are independent of breast-feeding at early age. Butyrate producers +vely correlated with BMI might indicate ↑ capability of energy harvest

Bervoets et al. 2013 [47]	Overweight and obese children (*n* = 26), healthy lean children (*n* = 27) age of 6–16 years	To assess differences in gut microbiota between lean and obese children	Selective plating and qPCR, MALDI-TOF-MS for detailed study of *Bacteroides fragilis* group. Dietary records for dietary intake	↑ F : B ratio in obese versus lean. ↓ *B. vulgatus* and ↑ *Lactobacilli *spp. in obese versus lean. In all groups, *Staph. aureus *+vely associated with energy intake. *Lactobacilli* in obese children +vely associated with plasma CRP	Obese microbiota are different from lean

Calvani et al. 2010 [49]	Morbidly obese (*n* = 15) and healthy lean (*n* = 10) adults	To assess differences in gut microbiota associated urinary metabolites between obese and lean and the effect of biliopancreatic or Roux-en-Y surgery on these metabolites	High-resolution proton NMR (1H NMR) spectroscopy	Baseline: ↓ levels of hippurate, xanthine, and trigonelline and ↑ levels of 2-hydroxybutyrate in obese versus lean. Inverse relationship of xanthine with plasma uric acids levels 3 months after surgery: reversal of the above metabolites with wt. loss	Obese phenotype is associated with a peculiar metabotype compared to lean. These metabolic changes are reversed with bariatric surgery

Druart et al. 2014 [60]	Obese women (*n* = 15)	To investigate the effect of prebiotic induced gut microbiota modulation on PUFA derived bacterial metabolites production	Inulin type fructans (oligofructose 50/50) supplementation (16 g/day) for 3 months, qPCR, human intestinal tract chip analysis, circulating fatty acids levels	Treatment with prebiotics did not affect levels of PUFA derived conjugated linoleic and linolenic acids. PUFA derived bacterial metabolites were −vely correlated with total cholesterol, LDL, and HDL, while they were +vely correlated with *Bifidobacterium *spp., *Eubacterium ventriosum*, and *Lactobacillus *spp.	

Fernandes et al. 2014 [29]	Overweight and obese adults (*n* = 37, age of 21–60 years), lean adults (*n* = 52, age of 18–67 years)	To investigate dietary intakes, faecal SCFA, gut microbiota composition, and physical activity levels in simple obese versus healthy lean adults	3-day food diary, breath methane and hydrogen, faecal SCFA, and qPCR	↑ acetate, propionate, butyrate, valerate, and total SCFA in obese versus lean. No difference in Firmicutes to *Bacteroides*/*Prevotella* ratio between lean and obese. ↑ *E. coli* in lean compared to obese. Irrespective of the group, total faecal SCFA were −vely correlated with *Bacteroides*/*Prevotella* and +vely correlated with Firmicutes/*Bacteroides* ratio	Obese phenotype carries distinct energy harvesting capability compared to lean. However, the evidence is not conclusive due to study limitations

Ferrer et al. 2013 [61]	Obese adolescent (*n* = 1), lean adolescent (*n* = 1)	To perform a holistic phylogenetic and functional analysis of the gut microbial communities of the lean and obese microbiome	454 FLX pyrosequencing, Orbitrap MS/MS	Lean microbiome more diverse than obese. High Firmicutes (~95% versus 78%) and low Bacteroidetes (~4% versus ~18%) in obese versus lean. Obese metagenome associated with vitamin B12 and 1,2-propanediol metabolism while lean metagenome associated with B6 metabolism. ↑ butyrate production in obese compared to lean	Lean and obese metagenome and microbiome differ from each other however; both show functional redundancies in terms of proteins expression

Karlsson et al. 2012 [62]	Overweight and obese (*n* = 20), lean (*n* = 20) children	To investigate differences in faecal gut microbiota between lean and obese children	qPCR and RFLP, liver function tests	↑ Enterobacteriaceae and ↓ *Desulfovibrio* and *A. muciniphila *in obese compared to lean. No difference in *Lactobacillus*, *Bifidobacterium*, and *Bacteroides fragilis* between lean and obese. Serum ALT −vely correlated with *Bifidobacterium. *No difference in faecal calprotectin between lean and obese	Differences in gut microbiota composition exist at an early age between lean and obese. The study is however cross-sectional. Not controlled for diet and based on PCR

Kong et al. 2013 [45]	Morbidly obese women (*n* = 30)	To assess the impact of Roux-en-Y gastric bypass surgery (RYGB) on the gut microbial population and its effect on the genes expression in white adipose tissue (WAT)	454 GS-FLX pyrosequencing of faecal samples at 0, 3, and 6 months after RYGB and dietary assessment	↑ Proteobacteria after RYGB by 37%, ↑ in association between 102 genera and 562 WAT genes. Bifidobacteria andFirmicutes such as *Dorea*, *Lactobacilli*, and *Blautia* ↓ and *Bacteroides* such as *Bacteroidetes* and *Alistipes* and Proteobacteria such as *E. coli* ↑ after 3 months. About 50% of changes in genes expression were independent of caloric intake. No difference seen between 3 and 6 months	Gut microbiota richness increases after RYGB with changes in association with genes expression in WAT. Further exploration of gut microbiota with weight loss is needed

Brignardello et al. 2010 [63]	13 obese and 11 normal weight adults	Evaluation of gut permeability in asymptomatic obese and its relationship with plasma and faecal markers of inflammation and alteration in gut microbiota	Lactulose- mannitol sucralose test for intestinal permeability, blood CRP, and fatty acids. Faecal G + C profiling, calprotectin, and leptin	CRP significantly ↑ in obese compared to nonobese. Faecal fat, calprotectin and leptin, and ARA/EPA not different in both groups. Obese subjects had ↑ in relative abundance bacteria with 23–37% G + C contents in their DNA and ↓ in the relative abundance of those with 40–47% and 57–61% of G + C content. G + C peak values −vely correlated with CRP values	Gut microbiota differ between obese asymptomatic and nonobese. ↑ CRP in asymptomatic obese individuals do not have signs of gut inflammation

*Studies suggesting the effect of diet on the gut microbiota and resultant obesity*					

Santacruz et al. 2010 [64]	16 overweight and 34 normal wt. pregnant women	To investigate the relationship between gut microorganisms, body wt., wt. gain, and various parameters in pregnancy	qPCR, blood glucose, total cholesterol, HDL, TG, LDL, urea, creatinine, uric acid, bilirubin, iron, ferritin, transferrin, folate, and food 24–72 h food diaries for caloric intake	Bifidobacteria and *Bacteroides* significantly ↑ and *E. coli* and *Staph aureus* ↓ in normal wt. Total bacteria especially *Staph. aureus* +vely correlated with cholesterol. *Lactobacillus* group −vely correlated with infant birth wt. in women with excess wt. gain	Bifidobacteria and *Bacteroides* may play a positive role in wt. management of pregnant women and in their metabolic regulation

Duncan et al. 2008 [65]	33 obese and 24 nonobese subjects	To examine the relationships between BMI, weight loss, and the major gut microbial groups	Gut microbiota quantification using FISH and quantitative PCR. Dietary intervention with high protein-low carbohydrate ketogenic diet and high protein moderate carbohydrate nonketogenic diet	No difference in total bacteria and *Bacteroides* between obese and nonobese. No significant relation between BMI, weight loss, diet order, and *Bacteroides*. ↓ *Roseburia-Eubacterium rectale.* ↓ Bifidobacteria after 4 weeks of low carbohydrate weight loss diets	No relationship of *Bacteroides* and Firmicutes ratio at phylum level with obesity

Duncan et al. 2007 [66]	20 obese healthy volunteers	To evaluate the effect of high protein and low fermentable carbohydrate diet on gut microbiota activity and population	Dietary intervention with maintenance, HPMC, and HPLC diets. Bacterial enumeration with FISH and butyrate with GC	Total SCFA ↓ during consumption of the HPMC and HPLC diets. Butyrate was ↓ for the HPLC compared to for the HPMC diet. Butyrate proportion ↓ as carbohydrate supply was ↓. Most abundant bacterial group was *Cytophaga-Flavibacterium-Bacteroides* group and *Clostridial cluster* IV. ↓ bacterial count, ↓ *Roseburia intestinalis* and *Eubacterium rectale*	Butyrate production and counts of certain bacteria are largely determined by the content of fermentable carbohydrate in the diet

Walker et al. 2011 [67]	16 obese stable weight subjects	To examine the influence of the precisely controlled diet on the human colonic microbiota population and composition	Intervention with maintenance diet, RS, NSP, low carbohydrate diet, and wheat bran. Chemical analysis of diet composition and digestibility. Real-time qPCR, denaturing gradient gel electrophoresis (DGGE)	Marked interindividual variation was noted. *Ruminococcus bromii ↑ *with RS diet. Oscillibacter group ↑ on the RS and WL diets. Relatives of *Eubacterium rectale* and *Collinsella aerofaciens* ↓ on WL	Different dietary carbohydrates can produce substantial changes in gut bacterial diversity

Schwiertz et al. 2010 [18]	30 normal weight, 35 overweight, and 33 obese adults	To evaluate the differences in gut bacteria and faecal short chain fatty acids between lean and obese individuals	Faecal samples for quantitative PCR and SCFA analysis	>20% higher SCFA in stools of obese than lean, with ↑ propionate and butyrate. Significantly ↑ *Bacteroides* in overweight compared to lean but not obese. +ve correlation between BMI and propionate, % propionate, Bifidobacteria and *Methanobrevibacter* even after correction for the influence of age and gender	Because of controversial results, no specific bacterial group can be attributed to obesity at this stage

Turnbaugh and Gordon 2009 [68]	31 adult mono- and 23 dizygotic (MZ and DZ) female twins and their mothers (*n* = 46)	To assess how gut microbiome is influenced by the host genotype, external environment, and the extent of host adiposity	UniFrac analysis, and gut microbiota assessed by 16SrRNA pyrosequencing	No significant difference in degree of similarity in gut microbiota of adult MZ versus DZ twin-pairs. ↓ *Bacteroides* and ↑ Actinobacteria in obese. No significant difference in Firmicutes. Phosphotransferases involved in microbial processing of carbohydrates rich in obese	Genomic profile of microbiota exists at a level of metabolic function and not by a definite set of microbiota

Jumpertz et al. 2011 [69]	12 lean and 9 obese adults	To assess influence of change in nutrient load on gut microbiota of lean and obese individuals and correlation of microbiota with energy harvest from diet	Stool and urine energy content with change in caloric content of diet, culture independent metagenomic studies of microbiota	Nutrient load caused 20% ↑ in Firmicutes and corresponding decrease in *Bacteroides* in lean subjects with approximately 150 kcal ↑ in energy harvest from diet	Nutrient load affects gut microbiota composition which is also associated with ↑ energy harvest from the diet

Weickert et al. 2011 [70]	Overweight and obese adults (*n* = 69, age of 24–70 yrs) with features of metabolic syndrome	To investigate mechanisms for the effect of high cereal fibre on insulin sensitivity by exploring gut microbiota composition and colonic fermentation	18 weeks of intervention with cereals. GC for SCFA. *In vitro* fermentation on healthy volunteer faeces with fibres, FISH, and flow cytometry. Euglycemic clamp for insulin sensitivity	No difference in faecal SCFA at 0, 6, and 18 weeks. No differences in SCFA with *in vitro* fermentation. *Roseburia* tended to ↓, *Clostridium cluster IX* ↓ after 6 weeks but not at 18 weeks, and *Atopobium* ↑ after 18 weeks. Insulin sensitivity improved after 18 weeks	Improvement in insulin sensitivity is not associated with colonic microbiota metabolism and fermentation

Cotillard et al. 2013 [71]	Obese (*n* = 38) and overweight (*n* = 11) adults	To investigate temporal relationship between food intake, gut microbiota, and metabolic and inflammatory phenotype	6-week energy restricted, high protein diet followed by 8 weeks of weight maintenance period, food diaries, and quantitative metagenomics	Gene counts showed bimodal distribution. Patients with low gene count (<480,000 genes) had a tendency towards ↑ LDL, dysmetabolism, insulin resistance, inflammation, and obesity and vice versa for high gene count. Weight loss diet partially ↓ inflammation and improves dysmetabolism but not to full extent	Obesity is associated with lower gene richness which is partially corrected by dietary intervention

Wt.: weight, GB: gastric bypass, OB: obese group, BMI: body mass index, TG: triglycerides, CRP: C-reactive protein, BCFA: branched chain fatty acids, qPCR: quantitative polymerase chain reaction, FISH: florescent *in situ* hybridization, F : B ratio: Firmicutes to *Bacteroides* ratio, +vely: positively, −vely: negatively, IL-6: interleukin-6, MALDI-TOF MS: matrix assisted laser desorption/ionization-time of flight mass spectrometry, RFLP: restriction fragment length polymorphism, NMR: nuclear magnetic resonance spectroscopy, ARA/EPA: arachidonic acid/eicosapentaenoic acid, HDL: high density lipoprotein, LDL: low density lipoprotein, HPMC: high protein, medium carbohydrate diet, HPLC: high protein-low carbohydrate diet, RS: resistant starch, NSP: nonstarch polysaccharide, WL: reduced carbohydrate weight loss diet, GC: gas chromatography, SCFA: short chain fatty acids, and TG: triglycerides.
